# Variation in clinical outcomes and process of care measures in community acquired pneumonia: a systematic review

**DOI:** 10.1186/s41479-020-00073-4

**Published:** 2020-09-25

**Authors:** H. Lawrence, W. S. Lim, T. M. McKeever

**Affiliations:** 1 Nottingham University Hospitals NHS Trust, Hucknall Road, Nottingham, UK; 2 Division of Epidemiology and Public Health, School of Medicine, Clinical Sciences Building, Nottingham City Hospital, University of Nottingham, Nottingham, UK; 3 NIHR Nottingham Biomedical Research Centre/Nottingham Clinical Research Facilities, Nottingham, UK

**Keywords:** Variation in healthcare, Community acquired pneumonia, Systematic review

## Abstract

**Background:**

Variation in outcomes of patients with community acquired pneumonia (CAP) has been reported in some, but not all, studies. Although some variation is expected, unwarranted variation in healthcare impacts patient outcomes and equity of care. The aim of this systematic review was to: i) summarise current evidence on regional and inter-hospital variation in the clinical outcomes and process of care measures of patients hospitalised with CAP and ii) assess the strength of this evidence.

**Methods:**

Databases were systematically searched from inception to February 2018 for relevant studies and data independently extracted by two investigators in accordance with the Preferred Reporting Items for Systematic Reviews and Meta-Analysis (PRISMA) statement. Included studies enrolled adults hospitalised with CAP and reported a measure of variation between two or more units in healthcare outcomes or process of care measures. Outcomes of interest were mortality, length of hospital stay (LOS) and re-admission rates. A structured synthesis of the studies was performed.

**Results:**

Twenty-two studies were included in the analysis. The median number of units compared across studies was five (IQR 4–15). Evidence for variation in mortality between units was inconsistent; of eleven studies that performed statistical significance testing, five found significant variation. For LOS, of nine relevant studies, all found statistically significant variation. Four studies reported site of admission accounted for 1–24% of the total observed variation in LOS. A shorter LOS was not associated with increased mortality or readmission rates. For readmission, evidence was mixed; of seven studies, 4 found statistically significant variation. There was consistent evidence for variation in the use of intensive care, obtaining blood cultures on admission, receiving antibiotics within 8 h of admission and duration of intravenous antibiotics. Across all outcome measures, only one study accounted for natural variation between units in their analysis.

**Conclusion:**

There is consistent evidence of moderate quality for significant variation in length of stay and process of care measures but not for in-patient mortality or hospital re-admission. Evidence linking variation in outcomes with variation in process of care measures was limited; where present no difference in mortality was detected despite POC variation. Adjustment for natural variation within studies was lacking; the proportion of observed variation due to chance is not quantified by existing evidence.

## Introduction

Geographical variation in clinical care is considered ubiquitous across all aspects of healthcare. A proportion of variation in healthcare measures is warranted, reflecting true differences in individual healthcare preferences and the needs of the local population served. Conversely, persistent unwarranted variation in clinical care directly impacts on equity of services, population outcomes and use of resources [1]. Equitable care across geographical regions has been highlighted as a key concern from a patient viewpoint [2]. Inevitably, outcome measures are increasingly used to rank healthcare between regions and hospital providers [3]. However, there is concern that such ranking does not account for natural variation between units and may not be reflective of true variation in quality of healthcare [4].

Community acquired pneumonia (CAP) remains a major cause of hospitalisation and mortality globally. In Europe it is estimated that the direct costs of pneumonia amount to 2.5 billion Euros per annum with the majority of this cost comprised of inpatient care [5]. Inter-hospital variation in outcomes of patients hospitalised with CAP was first suggested from retrospective claims-based studies in the USA [6]. More recent evidence from large GP databases in the UK have shown that mortality for patients under the age of 75 varies up to nine-fold depending on the geographical location [2]. Little is known about the causes of this apparent geographical variation, whether it extends to other outcomes or process of care measures and to what extent it is unwarranted.

The aim of this systematic review was to collate available evidence on regional and inter-hospital variation in the clinical outcomes and process of care measures of patients hospitalised with CAP and assess the strength of this evidence. Where possible, we also sought to identify any potential causes for any observed variation.

## Methods

We systematically searched online databases (MEDLINE, EMBASE, Web of Science) using Medical Subject heading (MeSH) terms to identify published and unpublished studies that compared the process of care measures and outcomes of adults hospitalised with CAP between two or more hospitals or geographical regions. As MeSH terms to identify variation excessively limited our search, we also broadened the search strategy to capture all studies on adults hospitalised with community-acquired pneumonia for title screening (Additional Material Appendix A). Databases were searched from inception to February 2018 inclusive. Title, abstract and full text screening were performed in a three-step process by two independent reviewers (HL, TM) using the online platform Covidence©. Disagreements were resolved by discussion and involvement of a third reviewer. Hand searching of references from the list of eligible studies for further references not identified in the initial search was performed. Data extraction was performed by each reviewer (HL, TM) independently using a standardised form. The review was conducted and reported in accordance with the Preferred Reporting Items for Systematic Reviews and Meta-Analysis (PRISMA) statement, [7] and prospectively registered on PROSPERO (CRD42019124068).

All prospective and retrospective observational or randomised controlled studies in any language with no date restriction on publication were considered for inclusion. Studies were included if they enrolled adults (> 16 years old) hospitalised with CAP and reported a measure of variation between two or more hospitals or geographically distinct areas in chosen outcome or process of care measures. For the purpose of this review, included studies defined CAP either; a) clinically as the acute onset of symptoms suggestive of lower respiratory tract infection with new infiltrates on thoracic imaging consistent with pneumonia or b) using recognised International Classification of Disease (ICD) codes pertaining to pneumonia from administrative databases. Geographical units for comparison were defined as geographical regions or geographically separated hospitals serving distinct patient populations. Measures of inter-hospital variability included appropriate descriptive statistics, variance analysis and graphical methods for comparing institutional performance.

Studies were excluded if: 1) they enrolled solely immunosuppressed patients with Human Immunodeficiency Virus (HIV) and Pneumocystis Pneumonia (PCP) 2) they enrolled patients exclusively from a primary care setting or 3) they examined temporal variation in CAP care only. Finally, studies that described or measured implementation of a change from usual care within a hospital setting, for example implementation of a pneumonia care pathway or an alternative antimicrobial regime, were also excluded.

Primary outcome measures of interest were case mortality, length of hospital stay and hospital re-admission rates. In accordance with recognised guidelines for the management of CAP, process of care measures of interest were: a) use of guideline adherent antibiotics; b) admission rates to intensive care units; c) duration of antibiotic treatment (both intravenous and total); d) time to first antibiotic and e) obtaining admission blood cultures [8, 9].

### Statistical analysis

Due to differences in the statistical methods used to evaluate variation across the included studies, a pooled meta-analysis was not possible. Instead, a structured synthesis of the studies was performed by collating: 1) inter-hospital ranges for outcome and process of care measures, 2) variance analysis and 3) statistical methods to quantify or control for natural variation between units.

### Assessment of Bias

Two reviewers (HL & TM) assessed the methodological quality of studies using a modified quality score based on the Newcastle-Ottawa Scale. This score assesses the risk of bias at outcome level in observational studies in 3 domains: participant selection, comparability of groups and validity of outcome domains. The maximum score on the modified scale used was 10.

## Results

Comprehensive searching identified 5738 papers. Following title and abstract screening, 88 studies were included in the full text assessment, from which 67 studies were excluded; the main reason for exclusion was the lack of reporting on variation (Additional Figure 1, additional material). One study was identified following hand searching of references and subsequently included in the review [10].

### Characteristics of included studies

Twenty-two papers met the inclusion criteria [10–31]. Results from two papers were derived from the same study population and were combined for further analysis [26, 28]. A further two papers reported results from the same population but different measures, so were both included [19, 25]. Details and characteristics of included studies with a description of variation between units compared, their respective populations and disease characteristics are shown in Table 1.

**Table 1:** Characteristics of Included Studies

Author & Year	Study Design	Country	Number of subunit compared	Total study population	Quality score	POC, Outcome or Both	Variation in Patient population between units	Variation in hospital type / subunit	Variation in disease factors
*Subunit of variation: Geographical region or country*									
*Arnold* et al. [12] 2013	Retrospective Cohort	International – 16 countries across USA, Canada, Europe and Latin America	70 hospitals across 3 geographical regions (USA/Canada, Europe, Latin America)	6371	9.5	Both	Significant differences in baseline populations. Latin America lowest prevalence of every co-morbidity.	Variation between hospitals grouped by continents. International variation in healthcare practice and resources.	Europe - fewest low severity scoring patients, greatest number of high severity scoring patients.
*Blasi* et al. [13] 2013	Retrospective Cohort	International - Europe	10 countries (128 sites)	2039	6.5	Outcome	Not reported	Not reported	Included HCAP in addition to CAP
*Lave* et al. [23] 1996	Retrospective Analysis of Administrative data	USA	7 geographical regions	36,222	7	Both	Not reported	All hospitals part of a larger non-profit organisation. Bed size varies 80–500 beds. Teaching and non-teaching facilities.	Not reported
*Remond* et al. [27] 2010	Mixed Prospective / Retrospective Cohort	Australia	2 regions (7 hospitals)	293	6.5	Both	Different ethnicity between cohorts	Six small regional hospitals in the Kimberley, one tertiary hospital in Central Australia	Regional differences in isolated causative organisms.
*Subunit of variation: Hospital*									
*Aelvoet* et al. [11] 2016	Retrospective Analysis of Administrative data	Belgium	111 hospitals	108,213	7	Outcome	Not reported	All hospitals in Belgium	Not reported
*Cabre* et al. [14] 2004	Retrospective Cohort	Spain	27 hospitals	1920	6.5	Both	The number of comorbidities varied among hospitals.	All community hospitals - urban and rural	Proportion of patients belonging to each risk class (by PSI) varied widely among hospitals
*Capelastegui* et al. [15] 2005	Retrospective Cohort	Spain	5 hospitals	1498	6	Both	Statistically significant differences in patient demographic factors between hospitals.	All teaching general hospitals with similar resources	Statistically significant differences in PSI score classification between hospitals
*Dedier* et al. [16] 2001	Retrospective Cohort	USA	38 hospitals	1062	5	Both	Not reported	All academic hospitals	Not reported
*Feagan* et al. [17] 2000	Retrospective Cohort	Canada	20 hospitals	858	6.5	Both	Only comparison reported between teaching and general hospital populations	11 teaching hospitals, 9 community hospitals	Not reported
*Fine* et al. [10] 1993	Prospective Cohort	USA	4 hospitals	552	9.5	Both	Mean number of comorbid conditions per patient varied significantly among hospitals.	2 university hospitals, one veterans hospitals, one community hospital	Disease severity and aetiology similar across hospitals
*Garau* et al. [18] 2008	Retrospective Cohort	Spain	10 hospitals	3233	8	Outcome	Not reported	All tertiary hospitals	Proportion of patients belonging to each PSI class varied widely across hospitals, as did the proportion with an aetiological diagnosis.
*Gilbert* et al. [19] 1998	Prospective Cohort	USA/Canada	4 hospitals	1328	9.5	Both	Significant differences in mean age, gender, racial distribution and comorbidities among the 4 sites.	Three university teaching hospitals, one community teaching	Statistically significant differences in causative organisms identified and severity of illness.
*Hedlund* et al. [20] 2002	Retrospective Cohort	Sweden	17 hospitals	982	5	Outcome		Seven university hospitals, 10 county hospitals.	The mean PSI varied between 0.9 and 1.9 at different sites
*Iroezindu* et al. [21] 2016	Prospective Case control	Nigeria	4 hospitals	400	6	Outcome	Not reported	All tertiary hospitals	Not reported
*Klausen* et al. [31] 2012	Retrospective Analysis of Administrative data	Denmark	22 hospitals	11,322	8.5	Outcome	Not reported	All Danish public health hospitals	Not reported
*Laing* et al. [22] 2004	Prospective Cohort	New Zealand	2 hospitals	474	7	Both	Similar demographics between the two populations except significant differences in ethnicity and rates of COPD.	“Similar institutions”	No significant differences in disease severity by PSI.
*Malone* et al. [24] 2001	Retrospective Cohort	USA	5 hospitals	330 (52 severe)	5.5	POC	Not reported	All acute care facilities (Centura)	Not reported
*McCormick* et al. [25] 1999	Prospective Cohort	USA/Canada	4 hospitals	1188	9	Both	A younger more mixed-race population identified at one site. The proportion admitted from a nursing home varied from 9 to 16%.	Three university teaching hospitals, one community teaching	Severity of illness and symptom profiles were similar across hospitals. One hospital had fewer “high risk” aetiology.
*Menendez* et al. [26] 2003	Prospective Cohort	Spain	4 hospitals	425	7	NA	Not reported	Not reported	Not reported
*Reyes Calzada* et al. [28] 2007	Prospective Cohort	Spain	4 hospitals	425	6	Both	No significant differences in co-morbidity, age and sex. Smoking significantly more frequent in two hospitals.	One tertiary and 3 district general hospitals	Not reported
*Schouten* et al. [29] 2005	Analysis of baseline population from RCT	Netherlands	8 hospitals	436	6.5	POC	Not reported	Eight medium sized hospitals in the south-east of the Netherlands	Not reported
*Sow* et al. [30] 1996	Prospective Cohort	France and New Guinea	2 hospitals	333	5	Outcome	Mean age and pre-existing illness rate was significantly lower in Guinea than France.	One hospital in the Republic of Guinea compared to one in France	Similar severity between cohorts (clinical definition not validated severity score)

Studies differed in design: seventeen were cohort studies (nine retrospective, seven prospective and one mixed), three were analyses of administrative data, [11, 23, 31] one was a case control study [21] and one study analysed the baseline population from a randomised control trial [29]. The median number of units compared across studies was five (IQR 4–15) with a median of 1022 (IQR 445–2009) cases of CAP. Four studies compared geographical regions, the remaining 18 compared hospitals. Retrospective cohort studies compared a greater number of units (range 3–38) than prospective cohort studies; the latter involved a maximum of four units.

The range of quality scores was 5–9.5 (mean 6.95, SD 1.45). The three commonest factors missing from the quality score were: no statement accounting for missing data, limited inter-hospital case-mix adjustment for clinical parameters or baseline characteristics and the absence of a financial or affiliation statement. Baseline characteristics of study cohorts were not always comparable. For example, two prospective cohort studies compared study populations with widely different health care resources and baseline characteristics [27, 30]. In addition, there were three international studies [12, 13, 30]; observed variation in these may reflect differences in international healthcare provision and use.

### Variation in outcome measures

**Fig. 1:**
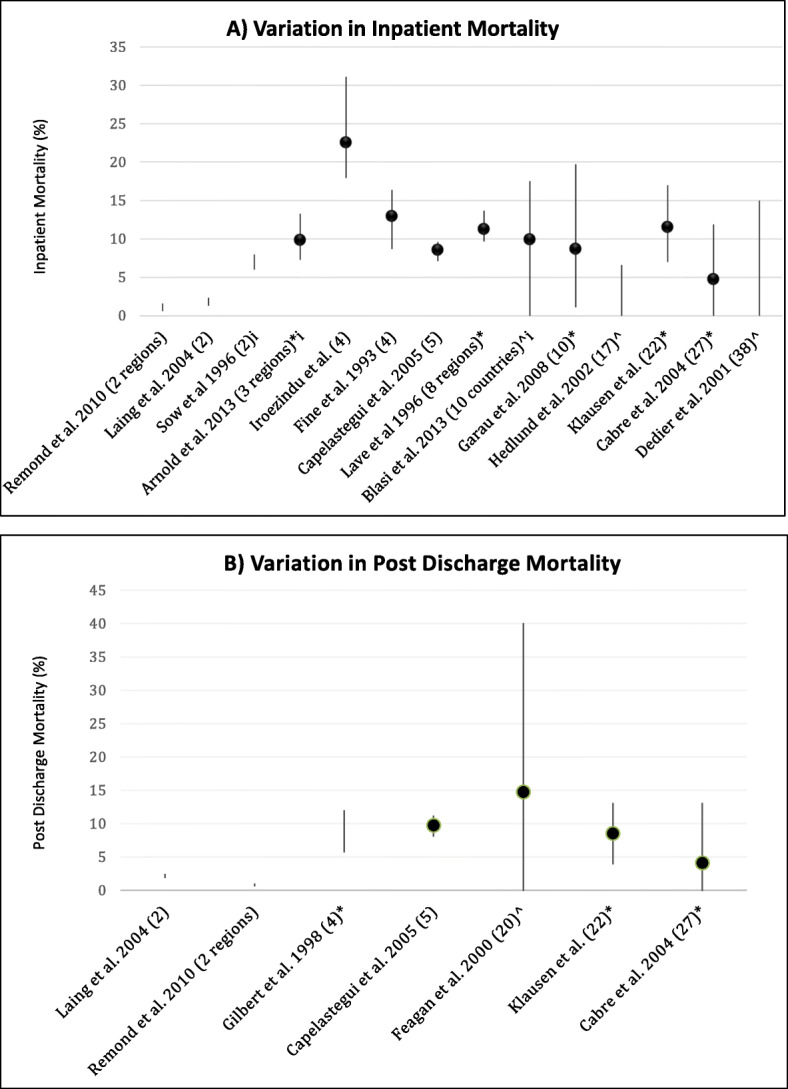
**a** Inter-hospital variation in inpatient mortality across studies (%). Range represented as line, dot represents mean value where possible. * denotes a statistically significant result. ^ denotes no reported p value. The letter ‘i’ represents an international study. The number in brackets represents the number of units compared in the hospitals, unless otherwise stated. **b**- Inter-hospital variation in post discharge mortality across studies (%) – 14 days post discharge or 30 days post admission. Range represented as line, dot represents mean value where possible. * denotes a statistically significant result. ^ denotes no reported *p* value. The number in brackets represents the number of hospitals compared in the study, unless otherwise stated

Fourteen studies reported variation in in-patient mortality [10, 12–16, 18, 20–23, 27, 30, 31]. The mean mortality for each study ranged between 1.1 and 22.6%. The magnitude of the observed range in variation for in-patient mortality was between 1 and 18.6% across studies (*n* = 14, mean 8.4%, SD 6.1). Of eleven studies that performed statistical significance testing, five found statistically significant variation (Fig. 1a and Additional Table 1) [12, 14, 18, 23, 31]. All 6 studies that did not find statistically significant variation in inpatient mortality compared 5 or fewer units [10, 15, 21, 22, 27, 30]. One study adjusted for natural variation between hospitals; *Aelvoet* et al. used the Spiegaelhalter method to produce funnel plots examining variation in standardised mortality ratios (SMRs) across 111 hospitals in Belgium [11]. Their primary model identified five institutions as ‘possibly better performing’, 7 as ‘possibly worse performing’ and 81 as ‘normally performing’ with the remaining 18 in an inconclusive ‘to be assessed’ category, with subsequent sensitivity analysis confirming these findings.

Mortality following discharge was reported by fewer studies (*n* = 8); one study reported mortality 14 days post discharge, [14] six reported 30-day post-*admission* mortality [15, 17, 19, 22, 27, 31] and one reported six-week mortality only [10]. Statistically significant differences were found in 3 of 7 studies that reported unadjusted *p* values (Fig. 1b) [14, 19, 31]. Three studies presented results adjusted for demographic and clinical variables [10, 19, 31]; one study identified sites with statistically significant higher mortality [31].

**Fig. 2:**
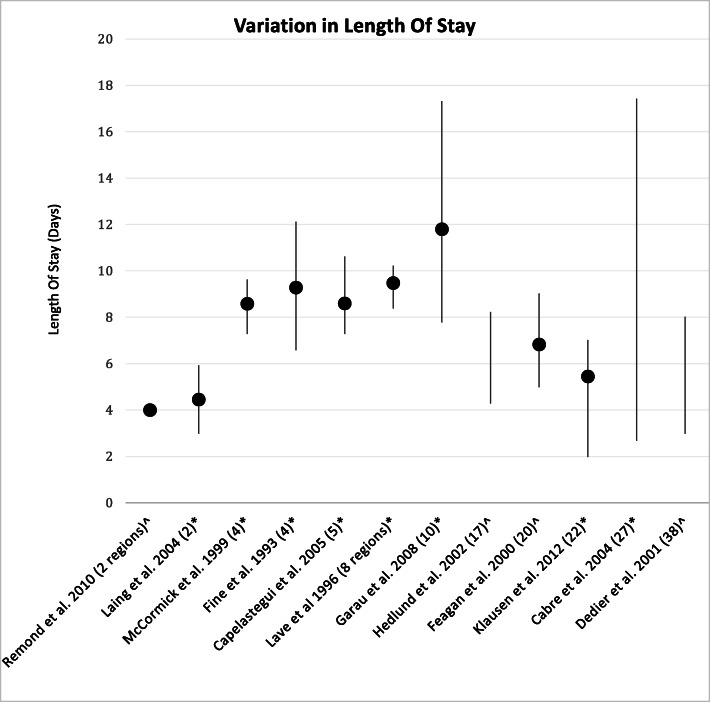
Inter-hospital variation in average LOS in days across studies where reported. Range represented as line, dot represents mean value where possible. * denotes a statistically significant result. ^ denotes no reported p value. The number in brackets represents the number of hospitals compared in the study, unless otherwise stated

Fourteen studies reported on variation in LOS [10, 14–18, 20–23, 25, 27, 28, 31]. The range in LOS variation (reported by 12 studies) was 0 to 14.7 days (mean 4.83 days, SD 3.89). Of nine studies reporting a range and *p*-value, all found statistically significant variation (Fig. 2 and Additional Table 1) [10, 14, 15, 18, 22, 23, 25, 28, 31]; six adjusted for confounders [10, 14, 15, 23, 25, 31]. One additional study reported in text that following adjustment for confounders the risk of having a LOS greater than the mean for the study population was significantly increased for two hospital sites by 2–3 fold [21].

The contribution of different factors towards variation in LOS was examined by 4 studies, each comparing inter-hospital variation (Table 2) [10, 14, 17, 22]. These studies were able to account for 21–61% of the total observed variation using statistical models adjusted for hospital site and different patient and disease characteristics. They found that the hospital of admission accounted for between 1 and 24% of the observed variation in LOS. The proportion of the total variation identified by each study due to hospital admission site (calculated as the variation accounted for by hospital site / the total variation accounted for by the model × 100) ranged between 1.6–41.7%. No study adjusted the results for natural variation. Laing et al. attributed 26% of the observed variation in LOS to process of care measures. Duration of intravenous antibiotics and admission to ICU were also significantly associated with LOS in that study [22].

**Table 2:** Contribution of hospital of admission to Variation in Length of Stay

Study	Number of hospitals	Adjusted for	P value	Total Variation accounted for by the model (%)	Variation accounted for by hospital site (%)	Proportion of total variation accounted for by the model due to hospital site (%)
*Cabre* et al. *2007* [14]	27	PSI risk class, Complications during hospitalisation, Admission to ICU, Oxygen therapy, Discharge to a NH	< 0.001	28.99	12	41.5
*Feagan* et al. *2000* [17]	20	PSI, Smoking status, COPD or asthma, Bacterial pneumonia	Not reported	21	7	33.3
*Fine* et al. *1993* [10]	4	PSI risk class, Age, NH resident, Race, Bacteraemia, Serum sodium <= 130 mmol/l, Hematocirt < 0.295, BUN > = 10.7 mmol/l	< 0.0001	24	10	41.7
*Laing* et al. *2004* [22]	2	Patient factors: Age, Duration of fever, COPD, PSI, Cerebrovascular disease, Complications of pneumonia, Heart failure, Ethnicity, Bacteraemia, Diabetes Process of care measures: Duration of IV antibiotics, Admission to ICU, Antibiotic guideline adherence, Macrolide and beta-lactam	< 0.01	61 Of which: Patient factors – 34 POC measures – 26	1	1.6

Four studies examined whether variation in LOS was associated with variation in other clinical outcomes; none reported significant findings [10, 14, 15, 25]. Specifically, a shorter LOS was not associated with increased mortality or readmission rates following multivariate analysis in two studies [14, 25]. No study examined post-discharge patient-reported outcome measures (PROMs) in relation to LOS.

**Fig. 3:**
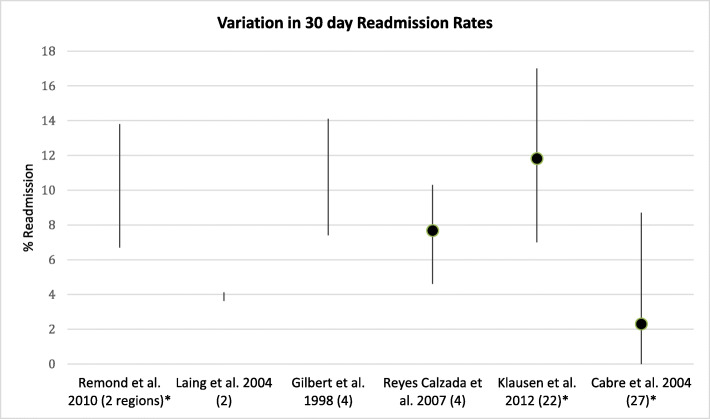
Inter-hospital variation in all cause readmission rates across studies. Range represented as line, dot represents mean value where possible. * denotes a statistically significant result. ^ denotes no reported p value. The number in brackets represents the number of hospitals compared in the study, unless otherwise stated. Data presented from McCormick et al. is 14-day post discharge readmission rates

Seven studies reported variation in the proportion of patients requiring re-admission for any reason [14, 19, 22, 25, 27, 28, 31]; two from the same study population reporting readmission at differing follow up points [19, 25]. Four found statistically significant differences (Fig. 3) [14, 25, 27, 31]. All results were unadjusted, except those from *Klausen* et al. who adjusted for gender, age, use of ventilatory support and Charlson index score, identifying three of 22 hospitals with increased re-admission rates [31].

### Variation in process of care measures

A wide range of process of care measures were reported across studies (Additional Table 2). Variation in the proportion of patients admitted to ICU (*n* = 7 studies) was found to be significantly different in the five studies that reported *p*-values for this outcome [10, 14, 15, 17, 23, 25, 27]. Similarly, significant variation was observed in the proportions with blood cultures obtained on admission (2 of 4 studies reported p-values; both *p* < 0.05) [12, 16, 27, 29]; receiving antibiotics within 8 h (2 of 4 studies reported p-values; both p < 0.05) [12, 15, 16, 29] and duration of intravenous antibiotics (4 of 5 studies reported *p* values; all 4 p < 0.05) [14, 15, 17, 19, 22]. Seven studies examined variation in adherence to antimicrobial guidelines (4 of 7 studies reported p-values; 2 reported p < 0.05) [15, 17, 22, 24, 27–29] while two studies examined variation in total antibiotic duration [15, 19]; one found statistically significant variation [15]. Laing et al. found significant variation in the duration of intravenous antibiotic therapy between two hospitals with no observed difference in mortality [22]. Following variance analysis they accounted for 41% of the observed variation in IV antibiotic duration, attributing 24% to patient characteristics, 4% to other management variables and 13% to hospital of admission (*p* < 0.001).

## Discussion

Of the three primary outcome measures of interest, we found consistent evidence for significant variation in relation to LOS, but not mortality or hospital readmission rates. There was consistent evidence for inter-hospital variation in all process of care measures examined, however evidence linking variation in outcomes with variation in process of care measures was limited.

The evidence for variation in LOS was consistent across studies and maintained following case-mix adjustment for patient and disease factors. Despite this, reasons for variation were not identified. Only one study was able to account for over 30% of the total observed variation [22]. Residual unaccounted variation may be attributed to i) unmeasured factors not included in the statistical models used or ii) natural variation due to chance. Multiple factors affect LOS, many of which were unmeasured within the studies (eg. physician behaviour, local healthcare system infrastructure) or competitively effect the direction of association (eg. better quality of care leading to survival of higher severity patients and ultimately a *longer* LOS). None of the studies used a statistical methodology to quantify or allow for natural variation in their analysis of LOS. Therefore, despite consistent evidence for variation, it is not possible to quantify what proportion is due to true differences between units rather than chance.

We observed significant variation in in-patient mortality only in larger studies comparing five or more units. Where variation in mortality was observed, care in the interpretation of results is warranted as adequate adjustment for both case-mix and natural variation were limited. In addition, none of the studies in this review adjusted for social deprivation; a recognised major determinant of inequalities in health, including mortality. In a UK community study, 80% of the regional variation in mortality from lower respiratory tract infections was accounted for by socio-demographic factors, as measured by the Index of Multiple Deprivation [32].

Only one study used a statistical method to control for the effect of natural variation when assessing variation in mortality; namely the Spiegaelhalter method used by *Aelvoet* et al. This method to identify outlying performing hospitals has been used elsewhere in national audit programmes to examine variation in healthcare [33]. It is an alternative method to reliability adjustment in removing the ‘chance’ element from the analysis of variation. As a graphical method for assessing variation in outcomes it has advantages over institutional ranking as it plots where institutions lie within the 95% (2 standard deviation) and 99.8% (3 standard deviation) predication limits around the mean. It can identify institutions that consistently lie outside these limits for further investigation. It incorporates the institutional sample size into the funnel plots as a measure of reliability of each institutional prediction. *Aelvoet* et al. identified providers with consistently outlying results within their single country study suggesting true variation in mortality from CAP. Outside this study, it is difficult to quantify from available evidence the proportion of observed variation in mortality that is due to true differences between units.

Outcome measures are increasingly used to rank institutions inevitably giving the appearance of ranking quality of care [4]. Rankability measures the proportion of the variation between providers with regards to an outcome that is due to true differences; it is considered high if above 70% [34]. No study in this review directly assessed the rankability of LOS as an outcome measure in CAP. The proxy measure generated in this review suggests a low rankability of < 50% across studies suggesting caution should be applied when making inferences about quality of care by ranking hospitals due to variation in LOS.

Although mortality is an important clinical outcome, it is a relatively infrequent outcome even in adults hospitalised with CAP; occurring in 10–15% of cases overall [35]. Small sample size and low event rates limit the statistical power to compare between hospitals [36]. Therefore, unless large sample sizes are obtained, mortality may be an insensitive marker to detect variation in care.

Many studies found CAP-related process of care measures to vary across hospitals. Evidence from observational studies suggests an association between selected clinical outcomes and certain process of care measures; a lower mortality has been associated with both earlier administration of antibiotics and obtaining blood cultures on admission while a decreased LOS has been associated with both antibiotic administration within four hours of admission and an appropriate switch from intravenous to oral antibiotics [37, 38]. However, none of the studies in this review were able to fully examine the association between variation in process of care measures and variation in clinical outcomes.

### Strengths and limitations

To overcome the lack of specific terminology identifying studies reporting on healthcare variation, we adopted a broad search criteria with additional hand searching of references to identify relevant studies. The quality of studies eligible for this review was moderate. However, due to inconsistencies in the statistical measures used across studies, meta-analysis was not possible and a structured synthesis was constructed. Reporting of the proportion of missing data and the subsequent handling of these data was absent in several studies, potentially reducing statistical power and introducing non-response bias to these studies.

Publication bias with studies observing minimal variation remaining unpublished is an important limitation. Such bias may account for the finding of variation in LOS in all relevant included studies. The majority of included studies were conducted in Europe or North America. Findings cannot be directly applied to health care systems in developing countries or other developed countries. Due to limited study numbers, results of studies reporting regional and inter-hospital differences were combined. Although limited to three studies, international differences in healthcare systems and populations served may bias results towards increasing observed variation.

### Implications

A key finding from this review is the need for more studies with robust methodologies to inform practice and policy in the future. The following recommendations are suggested:

- Future studies assessing the impact of healthcare variation in clinically important outcomes for patients hospitalised with pneumonia require large granular datasets comprising multiple subunits (at least 10, preferably > 20) each with representative patient samples.
- Datasets should include both process of care and detailed outcome data linked at a patient level. Process of care measures should include choice and duration of antibiotic therapy used.
- Linkage of datasets from multiple sources (eg. routinely collected hospital data, national audit data, primary care data) to allow rigorous case mix adjustment and assessment of the impact on healthcare following hospital discharge (re-consultation, further antibiotic prescription).
- Smaller exploratory studies of patient-centred outcome measures, such as PROMs, to assess the wider implications of variation from a patient perspective may be warranted.
- Robust and consistent statistical methodology that allows for natural variation should be used. The Spiegaelhalter method, increasingly used in the UK for national audit reports, is one suggested method [33, 39].
- Ranking of outcome measures should be avoided unless coupled with a valid assessment of rankability of the outcome measure utilised.

## Conclusions

In the management of adults hospitalised with CAP, there is consistent evidence of moderate quality for variation in LOS and process of care measures but not for in-patient mortality or hospital re-admission rates. Evidence linking variation in outcomes with variation in process of care measures was limited due to a lack of relevant studies. The proportion of observed variation due to chance is not quantified by existing evidence. This review highlights the importance of quantifying this in order to assess the validity of institutional (or regional) ranking by healthcare outcomes as a marker of quality of care in patients with CAP.

## Supplementary information

**Additional file 1.** Online Figure 1 Screening Consort Diagram. Table 1- Variation in Outcome Measures. Table 2 - Variation in Process of Care Measures. Additional Material Appendix A.

## Data Availability

All data generated or analysed during this study are included in this published article and it’s supplementary information files.

## Abbreviations

CAP: Community acquired pneumonia
COPD: Chronic obstructive pulmonary disease
ICD: International Classification of Disease
ICU: Intensive care unit
IQR: Interquartile range
LOS: Length of stay
MeSH: Medical Subject heading
PRISMA: Preferred Reporting Items for Systematic Reviews and Meta-Analysis
PROMs: Patient-reported outcome measures
SMR: standardised mortality ratios
