# Supplementary material for: Variation in clinical outcomes and process of care measures in community acquired pneumonia: a systematic review
Source: Pneumonia (Nathan). 2020 Sep 25;12:10. doi: 10.1186/s41479-020-00073-4 (PMC7517805; doi:10.1186/s41479-020-00073-4)
Supplement: Supplementary file 1 — Additional file 1. Online Figure 1 Screening Consort Diagram. Table 1- Variation in Outcome Measures. Table 2 - Variation in Process of Care Measures. Additional Material Appendix A. [file 41479_2020_73_MOESM1_ESM.docx]

# Additional Material:

## Additional Figure 1

591 Studies Imported

5738 Studies for Title and Abstract screening

88 studies full-text review

21 studies included

22 studies included

213 duplicates removed

5650 Excluded

67 Excluded

1 study found on hand searching references

**Online Figure 1- Screening Consort Diagram**

## Additional Table 1- Variation in Outcome Measures

| **Outcome** | **Study** | **Number of units** | **Inter-hospital range of outcome measure (% unless stated otherwise)** | **P value for difference between units** | **Mean (SD)** | **Adjusted?** | **Comments** |
| --- | --- | --- | --- | --- | --- | --- | --- |
| Average Length of Stay (days) | *Cabre et al. 2004(14)* | 27 hospitals | 2.7 – 17.4 (mean days) | 0.001 | 10.0 | PSI risk class, discharged to nursing home, admission to ICU |  |
|  | *Capelastegui et al. 2005(15)* | 5 hospitals | 7.3 – 10.6 (mean days) | <0.001 | 8.6 (1.25) | Adjusted analysis confirmed significant differences in mean LOS of up to 2.9 days (adjusted for PSI score, multi-lobar involvement, COPD and antibiotic treatment prior to admission). |  |
|  |  |  | 6 -10 (median days) | <0.001 | IQR 3 |  |  |
|  | *Dedier et al. 2001(16)* | 38 hospitals | 3 – 8 (median days) | Not reported | Not reported | - |  |
|  | *Feagan et al. 2000(17)* | 20 hospitals | 5 – 9 (median days) | Not reported | 6.83 (1.07) | - |  |
|  | *Fine et al. 1993(10)* | 4 hospitals | 6.6 – 12.1 (mean days) | <0.05 (Hospital D shorter mean LOS vs Hospitals A,B & C; Hospitals A,C&D each had shorter mean LOS than B) | 9.28 (2.25) | “Similar trend when adjusted by PSI” |  |
|  | *Garau et al. 2008(18)* | 10 hospitals | 7.8 -17.3 (mean days) | < 0.001 | 11.8 (2.79) | - |  |
|  | *Hedlund et al. 2002(20)* | 17 hospitals | 4.3 – 8.2 (mean days) | Not reported | Not reported | - | No correlation between mean PSI and mean LOS (p = 0.97) |
|  |  |  | 4.5 – 7 (median days) | Not reported | Not reported |  |  |
|  | *Iroezindu et al.(21)* | 4 hospitals | Not reported | Not reported | Not reported | - | On multivaritate analysis, predictors of LOS>10 days were care in hospitals A and B (aOR 3.1, 95% CI 1.3-10.5 and aOR 2.2, 95% CI 1.1-11.2 respectively) - adjusted for gender, CURB65, co-morbidity, anaemia, elevated Cr. |
|  | *Klausen et al.(31)* | 22 hospitals | 2 – 7 (median days) | Cox adjusted analyses identified 4 hospitals with shorter LOS (p<0.01) and 8 with longer LOS. | 5.45 (1.41) | Gender, age, ventilatory support, Chalson index score |  |
|  | *Laing et al. 2004(22)* | 2 hospitals | 3.0 - 5.9 (mean days) | <0.001 | 4.45 (2.05) | - |  |
|  | *Lave et al 1996(23)* | 8 regions | 8.4 – 10.2 (mean days) | <0.1 | 9.48 (0.53) | - | Differences in LOS across regions remained significant after controlling for severity of illness, aetiology and hospital characteristics (data not presented). |
|  | *McCormick et al. 1999(25)* | 4 hospitals | 7.3 – 9.6 (mean days) | <0.001 | 8.58 (1.03) | PSI risk class, Aetiology, ICU admission in first 24 hours, positive blood culture, discharge to NH, DNAR, employment status |  |
|  | *Remond et al. 2010(27)* | 2 geographic regions | 4 (median days) | Not reported | Presented as range | Not reported |  |
|  | *Reyes Calzada et al. 2007(28)* | 4 hospitals | Not reported | p=0.0001- hospital D median LOS compared to others | NA | - | "Shorter stays were recorded in hospital D, with a median of 6 days p=0.0001" |
| Inpatient mortality | *Arnold et al. 2013(12)* | 3 geographic regions | 7.3 – 13.3 | <0.0001 | 9.9% (3.08) | - |  |
|  | *Blasi et al. 2013(13)* | 10 countries | 0 – 17.5 | Not reported | 7.9% (6.04) | - |  |
|  | *Cabre et al. 2004(14)* | 27 hospitals | 0-11.9 | 0.012 p value for inter hospital difference | 4.8% | - |  |
|  | *Capelastegui et al. 2005(15)* | 5 hospitals | 7.1 – 9.6 | 0.94 | 8.62%, (1.01) | - |  |
|  | *Dedier et al. 2001(16)* | 38 hospitals | 0 - 15 | Not reported | Not reported | - |  |
|  | *Fine et al. 1993(10)* | 4 hospitals | 8.6 -16.4 | 0.32 | 13.0% (3.22) |  |  |
|  | *Garau et al. 2008(18)* | 10 hospitals | 1.1 – 19.7 | < 0.001 | 8.73% (5.49) | - |  |
|  | *Hedlund et al. 2002(20)* | 17 hospitals | 0 - 6.6 | Not reported | Not reported | - | No correlation between mean PSI and mean mortality rate (p=0.85) |
|  | *Iroezindu et al.(21)* | 4 hospitals | 17.9 – 31.1 | 0.53 | 22.58% (6.06) | - |  |
|  | *Klausen et al.(31)* | 22 hospitals | 7 – 17 | Identified 3 hospitals with higher IP mortality and one with lower (p <0.01) | 11.59% (2.67) | Gender, age, ventilatory support, Chalson index score | >65 years only |
|  | *Laing et al. 2004(22)* | 2 hospitals | 1.32 -2.35 | 0.8 | Presented as range | - |  |
|  | *Lave et al 1996(23)* | 8 regions | 9.7 – 13.7 | <0.1 for low and medium severity CAP | 11.31% (1.29) | - |  |
|  | *Remond et al. 2010(27)* | 2 geographic regions | 0.6 - 1.6 | >0.05 | 1.1% | - |  |
|  | *Sow et al 1996(30)* | 2 hospitals | 6 – 8 | >0.5 | 7% | - |  |
| Post discharge mortality | *Cabre et al. 2004(14)* | 27 hospitals | 0 – 13.0 | <0.001 | 4.1% | - | 14 days post discharge |
|  | *Capelastegui et al. 2005(15)* | 5 hospitals | 8.1 – 11.1 | 0.93 | 9.74% (1.14) | - | 30 days post admission |
|  | *Feagan et al. 2000(17)* | 20 hospitals | 0 – 40.0 | Not reported | 14.76% (9.03 | - | 30 days post admission |
|  | *Fine et al. 1993(10)* | 4 hospitals | 11.3-21.8 | 0.31 | 17.35 % (4.58) | PSI risk class, Age, NH resident, Race, Bacteraemia, Serum sodium <= 130 mmol/l, Hematocirt < 0.295, BUN >=10.7 mmol/l | 6 weeks post discharge |
|  | *Gilbert et al. 1998(19)* | 4 hospitals | 5.8 – 11.9 | 0.01 | Not reported | Study site not statistically significant predictor of 30-day mortality in multivariate logistic regression controlling for patient demographics, severity at presentation and baseline site differences. | 30 days post admission |
|  | *Klausen et al.(31)* | 22 hospitals | 4 – 13 | Two hospitals identified with higher mortality (p <0.01) | 8.54% (2.24) | Gender, age, ventilatory support, Chalson index score | >65 years only.  30 days post discharge |
|  | *Laing et al. 2004 (22)* | 2 hospitals | 1.97 – 2.35 | 0.5 | Presented as range | - | 30 days post admission |
|  | *Remond et al. 2010 (27)* | 2 geographic regions | 0.7 – 0.9 (30 day post admission) | >0.05 | Presented as range | - | 30 days post admission |
| Re-admission following discharge | *Cabre et al. 2004 (14)* | 27 hospitals | 0 – 8.7 | 0.004 | 2.3% | - | Unspecified |
|  | *Gilbert et al. 1998 (19)/ McCormick et al. 1999 (25)* | 4 hospitals | 7.4 – 14.1 | 0.08 | Not reported | - | 30 days post admission |
|  |  |  | 5 – 13 | 0.03 | 9% (3.65) | - | 14 days post discharge |
|  | *Klausen et al. (31)* | 22 hospitals | 7-17 | Effect parameterization in Cox regression analysis identified 3 hospitals with higher readmission rate (p <0.01) | 11.82% (2.46) | Gender, age, ventilatory support, Chalson index score | 30 days post discharge |
|  | *Laing et al. 2004 (22)* | 2 hospitals | 3.62 – 4.12 | 0.5 | Presented as range | - | 30 day readmission |
|  | *Remond et al. 2010 (27)* | 2 geographic regions | 6.7 – 13.8 (all cause) | <0.05 | Presented as range | - | 28 day readmission |
|  |  |  | 2.4 – 6.9 (respiratory) | >0.05 | Not reported | - |  |
|  | *Reyes Calzada et al. 2007 (28)* | 4 hospitals | 4.6 – 10.3 | 0.6 | 7.68% (2.36) | - | 30 days post discharge |

Additional Table 1 – Presented range of inter-hospital outcome measures across studies with p values and calculated mean and SD where possible. LOS- Length of Stay NA- not available

## Additional Table 2 - Variation in Process of Care Measures

| **Process of Care Measure** | **Study** | **Number of units** | **Inter-hospital outcome range (%)** | **P value for difference between units** | **Mean (SD)** | **Comments** | **Statistically significant variation in Outcome Measures reported?** |
| --- | --- | --- | --- | --- | --- | --- | --- |
| Admission to ICU (%) | *Cabre et al. 2004 (14)* | 27 hospitals | 0 – 10.7 | 0.002 | 3.3 |  | M, PDM, LOS, Readm |
|  | *Capelastegui et al. 2005 (15)* | 5 hospitals | 0 – 7.3 | 0.03 | 3.36, (2.61) | - | LOS |
|  | *Feagan et al. 2000 (17)* | 20 hospitals | 0.0 – 31.4 | Not reported | 13.33 (8.48) | - |  |
|  | *Fine et al. 1993 (10)* | 4 hospitals | 11.3 – 15.8 | Not reported | NA | - | LOS |
|  | *Lave et al 1996 (23)* | 7 regions | 14.4 – 18.1 | <0.01 | 16.49 (1.24) | - | M, LOS |
|  | *McCormick et al. 1999 (25)* | 4 hospitals | 6 – 25 | <0.001 | 14.5 (8.10) | ITU admission within the first 24 hours | LOS |
|  | *Remond et al. 2010 (27)* | 2 geographic regions | 1.8 – 23.8 | <0.001 | NA | - | Readm |
| Blood cultures obtained on admission (%) | *Arnold et al. 2013 (12)* | 3 geographic regions | 58 – 87 | <0.001 | 69.3 (15.5) | - | M |
|  | *Dedier et al. 2001 (16)* | 38 hospitals | 53.6 – 100 | Not reported | NA | Obtained within 24 hours |  |
|  | *Remond et al. 2010 (27)* | 2 geographic regions | 56.7 - 93.0 | <0.001 | As presented by range | - | Readm |
|  | *Schouten et al. 2005 (29)* | 8 hospitals | 48 -67 | Not reported | 57 (median) | 2 sets of cultures |  |
| Antibiotics within 8 hours of presentation (%) | *Arnold et al. 2013(12)* | 3 geographic regions | 71 - 86 | <0.001 | 80 (7.9) | - | M |
|  | *Capelastegui et al. 2005 (15)* | 5 hospitals | 59.6 - 84 | <0.001 | 70.8 (10.8) | - | LOS |
|  | *Dedier et al. 2001 (16)* | 38 hospitals | 53.8 – 100 | Not reported | NA | - |  |
|  | *Schouten et al. 2005 (29)* | 8 hospitals | 36 – 87 | Not reported | 68 (median) | Within 4 hours of presentation |  |
| Adherence to antimicrobial guidelines (%) | *Capelastegui et al. 2005 (15)* | 5 hospitals | 71.4 – 89.7 | <0.001 | 83.4 (7.3) | - | LOS |
|  | *Feagan et al. 2000 (17)* | 20 hospitals (ATS guideline compliance) | 47.9 - 100 | Not reported | 80.33 (14.0) | - | LOS |
|  | *Laing et al. 2004 (22)* | 2 hospitals | 47 - 66 | 0.02 | Presented as range | Severe cohort only |  |
|  | *Malone et al. 2001 (24)* | 5 hospitals | 16.7 -50 | Not reported | 30.7 (12.5) | Severe cohort only |  |
|  | *Remond et al. 2010 (27)* | 2 geographic regions | 16.3 – 28.7 | <0.05 | As presented by range | - | Readm |
|  | *Reyes Calzada et al. 2007 (28)* | 4 hospitals | 53.4 – 84.6 | 0.0001 | 72.52 (15.1) | - | LOS |
|  | *Schouten et al. 2005 (29)* | 8 hospitals | 5.0 - 59 | Not reported | 45 (median) | - |  |
| Duration of IV therapy (days) | *Cabre et al. 2004 (14)* | 27 hospitals | 2.5 - 6.9 (mean) | 0.001 | 4.6 (3.6) | - | M, PDM, LOS, Readm |
|  | *Capelastegui et al. 2005 (15)* | 5 hospitals | 3 – 7.9 (mean) | <0.001 | 5.3 (1.9) | - | LOS |
|  | *Feagan et al. 2000(17)* | 20 hospitals | 3.0 – 6.5 (median) | Not reported | 5.2 (0.9) | - |  |
|  | *Gilbert et al. 1998 (19)* | 4 hospitals | 6.0 – 7.0 (median) | 0.002 | 6.6 (0.58) | - | PDM |
|  | *Laing et al. 2004 (22)* | 2 hospitals | 1.7 – 3.0 (mean) | 0.001 | Presented as range | - | LOS |
| Total antibiotic duration (days) | *Capelastegui et al. 2005 (15)* | 5 hospitals | 12.9 – 16.4 (mean) | <0.001 | 14.4 (1.3) | - | LOS |
|  | *Gilbert et al. 1998 (19)* | 4 hospitals | 13.0 – 15.0 (median) | 0.49 | 14 (0.8) | - |  |

Additional Table 2 – Presented range of inter-hospital process of care measure achievement across studies with p values and calculated mean and SD where possible. LOS- Length of Stay NA- not available, M = Inpatient Mortality, PDM = post discharge mortality, Readm = Readmission

## Additional Material Appendix A:

| \| 1. exp PNEUMONIA/ or pneumonia.mp. \|  \| \| --- \| --- \| \| 2. exp Respiratory Tract Infections/ \|  \| \| 3. lower respiratory tract infection.mp. \|  \| \| 4. community acquired infection.mp. or exp Community-Acquired Infections/ \|  \| \| 5. exp "OUTCOME AND PROCESS ASSESSMENT (HEALTH CARE)"/ \|  \| \| 6. exp MORTALITY/ or mortality.mp. \|  \| \| 7. exp Hospitalization/ \|  \| \| 8. exp "Quality of Health Care"/ \|  \| \| 9. exp spatial analysis/ \|  \| \| 10. exp GEOGRAPHY, MEDICAL/ \|  \| \| 11. geographic varia*.mp. \|  \| \| 12. 1 or 2 or 3 \|  \| \| 13. 4 and 12 \|  \| \| 14. 5 or 6 or 7 or 8 \|  \| \| 15. exp "Length of Stay"/ \|  \| \| 16. 14 or 15 \|  \| \| 17. 13 and 16 \|  \| \| 18. limit 17 to "all adult (19 plus years)" \|  \| \| 19. exp RESPIRATORY TRACT DISEASES/ \|  \| \| 20. 9 or 10 or 11 \|  \| \| 21. 19 and 20 \|  \| \| 22. limit 21 to "all adult (19 plus years)" \|  \| \| 23. 18 or 22 \|  \| \| 24. 17 or 21 \|  \| |
| --- | --- | --- | --- | --- | --- | --- | --- | --- | --- | --- | --- | --- | --- | --- | --- | --- | --- | --- | --- | --- | --- | --- | --- | --- | --- | --- | --- | --- | --- | --- | --- | --- | --- | --- | --- | --- | --- | --- | --- | --- | --- | --- | --- | --- | --- | --- | --- | --- |
